# Mechanisms of troponin release into serum in cardiac injury associated with COVID-19 patients

**DOI:** 10.46439/cardiology.1.006

**Published:** 2021-03-08

**Authors:** R. John Solaro, Paola C. Rosas, Paulina Langa, Chad M. Warren, Beata M. Wolska, Paul H. Goldspink

**Affiliations:** 1 Department of Physiology and Biophysics, University of Illinois at Chicago, Chicago, IL, USA; 2 Center for Cardiovascular Research, University of Illinois at Chicago, Chicago, IL, USA; 3 Division of Cardiology, College of Medicine, University of Illinois at Chicago, Chicago, IL, USA

**Keywords:** COVID-19, Troponin, Myocardial injury, Hypertrophic cardiomyopathy

## Abstract

Serum levels of thin filament proteins, cardiac troponin T (cTnT) and cardiac troponin I (cTnI) employing high sensitivity antibodies provide a state-of-the art determination of cardiac myocyte injury in COVID-19 patients. Although there is now sufficient evidence of the value of these determinations in patients infected with SARS-CoV-2, mechanisms of their release have not been considered in depth. We summarize the importance of these mechanisms with emphasis on their relation to prognosis, stratification, and treatment of COVID-19 patients. Apart from frank necrotic cell death, there are other mechanisms of myocyte injury leading to membrane fragility that provoke release of cTnT and cTnI. We discuss a rationale for understanding these mechanisms in COVID-19 patients with co-morbidities associated with myocyte injury such as heart failure, hypertension, arrythmias, diabetes, and inflammation. We describe how understanding these significant aspects of these mechanisms in the promotion of angiotensin signaling by SARS-CoV-2 can affect treatment options in the context of individualized therapies. Moreover, with likely omic data related to serum troponins and with the identification of elevations of serum troponins now more broadly detected employing high sensitivity antibodies, we think it is important to consider molecular mechanisms of elevations in serum troponin as an element in clinical decisions and as a critical aspect of development of new therapies.

## Introduction

In the early phases of the pandemic caused by the SARS-CoV-2 virus (severe acute respiratory syndrome coronavirus 2) the emphasis of diagnosis and treatment was on acute respiratory distress syndrome (ARDS). Moreover, early guidelines from the American Heart Association (AHA) advised against determinations of serum cardiac troponin I (cTnI) and cardiac troponin T (cTnT), which are universally accepted biomarkers of myocardial injury [1]. Cardiac troponin (cTn) is a regulatory protein complex consisting of three units located at the sarcomere thin filament [2]. An inhibitory unit (cTnI) and a tropomyosin binding unit (cTnT) are responsible for maintaining a relaxed state when intracellular Ca^2+^ concentrations are low in diastole. In systole, there is a rise in intracellular Ca^2+^ that binds to cardiac troponin C (cTnC) releasing inhibition and promoting contraction and ejection. cTnI and cTnT are unique in cardiac myocytes, whereas cTnC is also expressed in slow skeletal muscle [1]. We previously reviewed mechanisms related to the complex biology of cTn leading to release of cTnI and cTnT to serum in a variety of clinical conditions, in the broad population, and in strenuous exercise [1]. In a paper responding to the AHA guidelines regarding use of these biomarkers in COVID-19 patients, Chapman et al. [3] published a statement of strong support for the value of determinations of serum cTnI and cTnT as biomarkers for cardiac injury in SARS-CoV-2 infected patients, who are older with coronary syndromes, demand ischemia, and inflammation. Chapman et al. [3] emphasized the value of determination of serum troponins as a way of evaluating the use of inotropic agents, vasopressors, and diuretics in COVID-19 patients. In our previous review [1], we also discussed briefly how the relation between the complex biology of cTn and the use of high-affinity cTnI and cTnT antibodies provide a rationale for the general idea that measures of serum troponins are useful diagnostic markers in COVID-19 patients. More recently, Sandoval et al. [4] reported an in-depth review of the literature showing that detection of elevated cTnI and cTnT in serum is a common occurrence in patients with severe COVID-19 disease. They emphasized the susceptibility and likelihood of cardiac injury in patients with severe COVID-19 disease, who demonstrate ARDS and associated inflammatory, prothrombotic, and procoagulant responses, leading to myocardial infarction, sepsis, and heart failure. Sandoval et al. [4] also described an approach for risk stratification employing serial troponin biomarker measurements together with other biomarkers. Gómez Antúnez et al. [5] have published an example of the presence of co-morbidities leading to increase mortality of patients with chronic obstructive pulmonary disease (COPD) hospitalized with SARS-CoV-2 infection. Cardiovascular related comorbidities noted were hypertension, atrial fibrillation, heart failure, ischemic heart disease, peripheral vascular disease, hyperlipidemia, diabetes mellitus and kidney failure. Moreover, Scarl et al. [6] recently reported a significant correlation between elevations of serum cTnI and markers of inflammation with mortality of patients hospitalized with SARS-CoV-2 infection, who demonstrated co-morbidities of pre-existing cardiovascular diseases, hyperlipidemia, and ARDS.

Even though the contributions described above are valuable in the clinical setting, mechanisms by which cTnI and cTnT appear in serum in SARS-Co-V-2 infections are generally not discussed except in general terms. We think it is important and informative to discuss these mechanisms. Our aim here is to provide clearer understanding of the molecular mechanisms responsible for release of cTnI and cTnT from cardiac myocytes in SARS-CoV-2 infected patients. Moreover, there is recent evidence that SARS-Co-V-2 can directly infect cardiac myocytes, but potential mechanisms leading to release of cTnI and cTnT have not to our knowledge been discussed [7,8]. We think this understanding is a critical aspect of evidence-based stratification of patients. As emphasized in our recent review [1] and discussed here, death of cardiac myocytes in COVID-19 patients is a complex process occurring not only by frank necrosis, but also by programmed cell death associated with inflammation. Membrane instabilities are also associated with mechanical stresses involving interactions among elements in the extracellular matrix and cytoskeletal network [9] that affect the barrier function of the phospholipid bilayer of the sarcolemma. With the wider use of the tools of “omics” such as genomics, proteomics, metabolomics. in precision medicine, it is inevitable that there will be discoveries necessitating in depth understanding of the molecular mechanisms in the myocyte responsible for elevations in serum cTnI and cTnT. Here, we discuss relevant examples of the how this understanding can influence clinical decisions as well as provide leads to discovery of therapeutic approaches. We first describe mechanisms of elevations in cTnI and cTnT serum levels and the relation of these mechanisms to COVID-19 patients.

## Mechanisms of cTn Release from Cardiomyocytes in COVID-19 Patients

**Figure 1:**
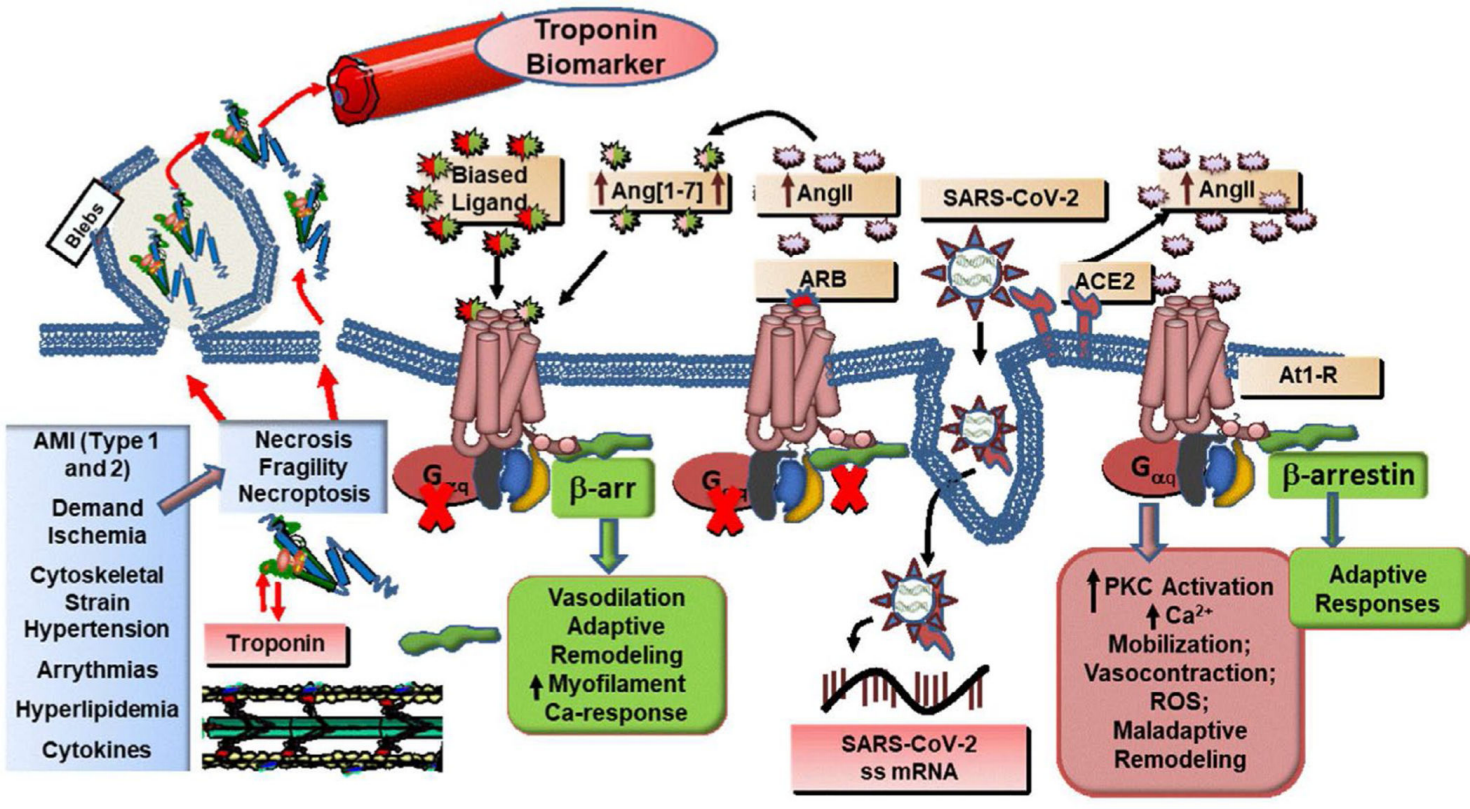
A hypothesis for mechanisms of release of cardiac troponins into serum in COVID-19 patients. Current evidence indicates that SARS-CoV-2 infects cardiac myocytes by binding to ACE2 and entering the cell by endocytosis and eventually releasing single-stranded mRNA that replicates. Lower levels of ACE2 induce an increase in AngII levels, an increase in binding to the AT_1_R, and activation of Gq and β-arrestin signaling (shown in green). Signaling *via* Gq induces maladaptations whereas signaling via β-arrestin not only desensitizes the receptor but also induces adaptive responses. With the elevation in AngII, there is generation of Ang [1–7], as a cardioprotective biased ligand favoring β-arrestin signaling over the Gq pathway. Also noted is evidence that with activation of β-arrestin signaling either by AngII or biased ligands there is an induction of its translocation directly to the sarcomeres thick and thin filaments illustrated in the lower left, as well as indicated in a cytoplasmic pool of cTn. Together with co-morbidities listed in the left-most panel, the dysfunctional AngII signaling arising from infection with SARS-CoV-2 leads to frank necrosis or membrane instabilities in which cTn is released from the cytoplasmic and likely the sarcomeric pool to the serum. AMI: Acute Myocardial Infarction; ROS: reactive oxygen species; ss mRNA: single stranded mRNA; AngII: Angiotensin II; ARB: Angiotensin Receptor Blocker; ACE2: Angiotensin Converting Enzyme 2; PKC: Protein Kinase C; See discussion in text and reference [1] for further information.

Figure 1 illustrates mechanisms connecting cardiac sarcomeres, the source of serum cTn, and membrane damage and fragility in cardiac myocytes of patients infected with the SARS-CoV-2. The left end of Figure 1 lists mechanisms leading to membrane fragility, bleb formation, rupture, and frank necrosis, which can lead to the release of cTn components that are in rapidly and slowly exchangeable pools in the myocyte. The left of Figure 1 lists mechanisms provoking release of cTnI and cTnT relevant to COVID-19 patients, especially the elderly, those with cardiovascular disease (CVD) including AMI, hypertension, arrythmia, demand ischemia including coronary disease, pro-thrombotic and pro-thrombosis events, and ARDS, obesity including hyperlipidemia and diabetes, and presence of pro-inflammatory cytokines. We next discuss how these diverse disorders lead to elevations in serum cTnI and cTnT.

Frank necrosis as the cause of myocardial injury appears as the common interpretation of induction of cTnI and cTnT release into serum in COVID-19 patients. This is understandable in view of the prevalent use of cTnI and cTnT as biomarkers to establish myocardial injury in patients presenting with Figure 1, in Type 1 AMI there is plaque rupture with ischemia inducing a loss of adenosine triphosphate (ATP), cell swelling, bleb formation and rupture, releasing cTnI and cTnT to the serum [10,11]. This loss of ATP and membrane blebbing may occur with SARS-CoV-2 infections associated demand ischemia and ARDS associated hypoxia, but other alterations need consideration. Membrane fragility with cTnI and cTnT release may occur with Type 2 MI, which involves a mismatch between oxygen demand and oxygen supply without plaque rupture [1]. This is a likely mechanism for the elevations in serum cTnI and cTnT levels in COVID-19 patients in which there is evidence of micro-circulatory changes with a propensity to thrombosis and coagulation abnormalities inducing hypoxia [12,13]. A result of the hypoxia is the inefficient production of ATP leading to the generation of reactive oxygen species (ROS), which is one of the mechanisms that affect stability of the sarcolemma by modifying proteins of the cytoskeletal network responsible for stabilization of the phospholipid bilayer.

As discussed extensively in our earlier review, inclusion of the cytoskeletal network mechano-transduction and myocyte sarcolemma stability provides a mechanism for the elevations of serum cTnI and cTnT in hypertension, heart failure, arrhythmias, exercise, and inotropic interventions [1]. These instabilities are likely to occur in COVID-19 patients, with heart failure, hypertension, and arrhythmias, which stress the myocardial cytoskeletal network, and which have been correlated with cTnI and cTnT release [14,15]. The integrity of the myocyte sarcolemma depends on the stability of the phospholipid bilayer depicted in Figure 1. There is ample evidence that the promotion of membrane instabilities is related not only to post-translational modifications of the network, for example in oxidative stress, but also to adverse mechanical forces involving “outside in” and “inside out” signaling across the myocyte cell membrane. Outside in signaling from the extracellular matrix to the intracellular cytoskeletal network maintains phospholipid and membrane stability in homeostasis, but with stresses there is a loss of this stability. Adverse signaling is associated with disorders that stretch the myocardium such as heart failure, hypertension and arrhythmias. Membrane instabilities may also arise from “inside out” mechanical strain affecting the cytoskeletal/integrin network, for example, the increased contractility induced by inotropic agents and as discussed below, the hypercontractility in hypertrophic cardiomyopathy (HCM).

Non-necrotic programmed myocyte cell death (necroptosis) is likely an important mechanism in COVID-19 patients leading to membrane fragility, but generally not considered. Unlike frank necrosis, necroptosis involves signaling cascades that can be up and downregulated [9,16]. Necroptosis is distinct from apoptosis. Apoptosis involves proteolysis and disposal of cellular constituents and is not likely to lead to release of cTn into the serum. A discussion of necroptosis is beyond the scope of this commentary, but it should be noted that elements in the pathway may be useful as serum biomarkers [1]. In addition, Guzzi et al. [17] reported an analysis of the interactome of SARS-CoV-2 with human proteins and demonstrated evidence for modification in cell death pathways, mitochondrial mechanisms together with interaction and downregulation of the angiotensin converting enzyme receptor 2 (ACE2) membrane receptor discussed below. In one arm of the necroptosis pathway, there is destabilization of membrane stability likely to occur because of a modification of the integrin/cytoskeletal network. In a second arm of the pathway, there is opening of the mitochondrial permeability transition pore leading to cell death. Relevant to the cytokine storm in COVID-19 patients is evidence that the cascade in necroptosis is induced by interactions with TNFα, known to increase with AMI and coronary artery disease [18,19].

Scarl et al. [6] reported a correlation between hyperlipidemia, elevated serum cTnI levels and morbidity of hospitalized COVID-19 patients. A role for hyperlipidemia, which was also a significant co-morbidity in COVID-19 patients reported by Gómez Antúnez et al. [5], is well established in the literature to be associated with cardiac myocyte cell death in human diabetes [20]. Animal models of diabetes and high fat-induced obesity commonly demonstrate pathologies leading to elevations in serum levels of cTnI and cTnT including inflammation with IL-6 and tumor necrosis factor alpha (TNF-α), and inefficient generation of ATP resulting in increased ROS production, reduced contractility, ischemia, and increased demand ischemia [21,22]. Reversal of the changes induced in serum cTn and pathologies also occurred in diabetic models treated with Azilsartan, a blocker of the angiotensin receptor.

## SARS-CoV-2, Angiotensin, and Elevated Serum cTnI and TnT

We next discuss induction of cardiac membrane fragility in cardiac myocytes because of SARS-CoV-2 binding to the angiotensin converting enzyme (ACE) receptor, which is the well-documented triggering event in the infection [23,24]. Associations with the cardiovascular system related to the interaction with the ACE2 receptor comes from single nuclei RNA sequencing revealing that in common cardiac disorders such as aortic stenosis and heart failure with reduced ejection fraction (HFrEF) there was a preponderance of elevated expression of ACE2 in cardiac myocytes, with lower levels elevated in endothelial cells and fibroblasts [25,26]. Interestingly an increase in expression of ACE2 was seen in pericytes, which have been reported to be important in the no-reflow phenomenon by reducing coronary flow following ischemia [27]. Kuba et al. [28] and Liu et al. [29] reported that SARS-CoV-2 binding to ACE2 reduces serum ACE2 and increases serum angiotensin II (AngII) levels, which is illustrated in Figure 1. This increase in AngII engages a cascade *via* the G-protein-coupled receptor, AngII type 1 receptor (AT_1_R) that results in maladaptive Gq signaling promoting several pathological mechanisms in the heart including oxidative stress, hypertrophy/remodeling, fibrosis, vasoconstriction leading to hypoxia, ischemia, demand ischemia, and cytoskeletal strain [30–32]. There are also effects on fluid retention and blood pressure that affect cardiac homeostasis. As shown in Figure 1, AngII at the AT_1_R also activates a parallel adaptive signaling pathway via β-arrestin. Apart from its action in desensitizing AT_1_R, β-arrestin has additional effects acting as a scaffold for signaling molecules that are adaptive and include vasodilation, increased contractility [24–25], adaptive remodeling, cytoskeletal reorganization, increased myofilament Ca-response [24], and anti-apoptotic mechanisms [33]. In addition to β-arrestin signaling, another proposed beneficial consequence of the elevation in AngII is an increase in generation of angiotensin [1–7], which has been reported to act as a natural biased ligand promoting activity of β-arrestin, while competing for binding at the AT_1_R with AngII [34, 35]. The balance between these maladaptive and adaptive effects triggered by binding SARS-CoV-2 to ACE2 determines the extent to which the signaling pathways will provoke membrane necrosis or membrane fragility leading to release of troponins to the serum. This maladaptive signaling can be blocked with angiotensin receptor blocker (ARBs), but there has been controversy regarding their use, for example in COVID-19 patients with hypertension [36].

In a related therapeutic approach, Manglik et al. [37] proposed to treat patients infected with SARS-CoV-2 with biased ligands acting at the AT_1_-R. In contrast to biased ligands, unbiased ARBs block both arms of the downstream signaling from the AT_1_R via Gq (maladaptive signaling) and β-arrestin (adaptive signaling). Biased ligands, notably TRV 027 (Sar-Arg-Val-Tyr-Lys-His-Pro-Ala-OH) developed by Trevina, Inc, Chesterton, Pa, act as an ARBs but promote β-arrestin signaling. Thus, it is understandable to consider the use of biased ligands as an alternative to ARBs. Ang [1–7] is a natural biased ligand generated by elevations in Ang II as a consequence of ACE2-SARS-CoV-2 interactions. Recent studies have shown strong evidence for binding of Ang [1–7] to the AT_1_-R, where it acts as a natural cardioprotective biased ligand [34,35]. However, the actions of Ang [1–7] as an endogenous biased ligand are relatively weak compared to exogenous synthetic biased ligands. We have reported that long term treatments with a biased ligand in a model of genetic dilated cardiomyopathy showed cardio-protection superior to treatment with an ARB, losartan [38]. The biased ligand TRV 027 has been proposed by Manglik et al.[37] to treat patients infected with SARS-CoV-2, despite the failure of this treatment to meet clinical end points in a 30-day clinical trial (Blast-AHF) in heart failure patients. This proposal was based on further examination, summarized by Cotter et al. [39] on the long-term effects of treating patients with relatively high baseline systolic blood pressure with a lower dose than employed in the BLAST-AHF trial. This extended analysis revealed that TRV027 treatment was able to improve renal function in this group and reduce all-cause mortality at 180 days compared to the controls. Manglik et al. [37] propose that biased ligands have anti-inflammatory, anti-apoptotic, and vasodilatory effects that should be effective in SARS-CoV-2 infected patients. Although broad understanding of β-arrestin signaling and effects of biased ligands are complex and poorly understood, TRV027 has been shown to be safe. Trevena has announced a clinical trial with treatment of older hospitalized COVID-19 patients with TRV027 [40]. The focus is on ARDS with an endpoint of improvements assessed by biomarkers of the coagulation cascade. In the present paper, we relate the studies summarized above to the proposed use of biased ligands operating as ARBs and as promoters of β-arrestin signaling in patients infected with SARS-CoV-2.

We think the principles of precision medicine apply in the use of biased ligands in COVID-19 disease in patients with suspected increases in sarcomere response to Ca^2+^ [41,42]. In our study of the effects of biased ligands, we reported evidence that β-arrestins activated by TRV067 translocate directly to the myofilaments colocalizing with myosin heavy chains, elevating myosin light chain phosphorylation, and increasing the myofilament response to Ca^2+^ [38]. We hypothesize that this interaction promotes the force generating actin-myosin interaction by activating a cascade resulting in myosin light chain 2 phosphorylation, which is known to sensitize myofilament to Ca^2+^ [43]. It appears important in the use of biased ligands in patients with SARS-CoV-2 infections to screen for HCM since a further increase in sarcomere Ca^2+^ response may be detrimental. Small molecule sarcomere inhibitors are an emerging therapy for HCM [44]. An approach, described in our recent review [1], is to determine not only the levels of serum cTnT and cTnI in diagnosis of myocardial injury in HCM, but also to determine the state (proteolysis, post-translational modifications) of these proteins in blood. In any case, data demonstrating the role of the AT_1_R signaling in patients infected with SARS-CoV-2 led to the proposal to employ AT_1_R blockers (ARBS) in treating COVID-19 patients. Whether blocking the receptor with traditional ARBS is beneficial has been controversial, but it is apparent that patients with cardiovascular disease should continue to take angiotensin converting enzyme inhibitors and ARBs as indicated. A consensus in guidelines provided by several societies and summarized by Sommerstein et al. [36] is that it is important to continue treatment of heart failure, which is a strong co-morbidity in SAR-CoV-2 patients, especially in older patients. We next discuss further the implications of understanding mechanisms of troponin release to serum in practical clinical settings.

## Clinical Implications and the Importance of Considering Mechanisms of Troponin Release in SARS-CoV-2 Infected Patients

As summarized above, it is now recognized that an important clinical objective in patients infected with SARS-CoV-2 is to determine the level of myocardial injury at presentation and during disease progression [3,4,6]. Mortality is well correlated with hospitalized patients who have myocardial injury as determined by serum cTnI and cTnT levels. Some understanding of the injury can be obtained from serial determinations of serum cTnI and cTnT. Increasing levels (deltas) indicate an acute injury, whereas steady high levels indicate chronic injury. One of the unexpected outcomes of the studies on the role of cardiac injury in SARS-CoV-2 infections is appreciation of the significance of cTnI and cTnT serum measurements in disorders beyond AMI. Advancements in the use of these biomarkers in these disorders has occurred largely related to the development of highly sensitive antibodies and protocols for the timing and repetition of the measurements. With the development of these high sensitivity antibodies, detection of elevated serum troponins became more commonly associated with disorders beyond AMI [1,45]. However, how the troponins appear in serum has been described in general terms with no illumination of molecular mechanisms. For example, Chapman et al. list the disorders promoting cTn release into serum in COVID-19 patients as follows: AMI, tachyarrhythmias, stress cardiomyopathy, coronary microvascular ischemia, and viral myocarditis [3]. Although they also included a list of potential mechanisms giving rise to increases in serum troponins in these disorders (ACE-2, cytokine storm, adrenergic stimulation, coagulopathy, hypertension, and hypoxia), insights into molecular mechanisms in the myocyte were not provided.

Why is understanding mechanisms of elevations of serum cTn in COVID-19 important? Scholarly consideration of molecular mechanisms has become an essential element in the approaches of precision medicine and individualized therapies [41,42,46]. As indicated above, omic measurements demand molecular understanding. In the case of determination of serum cTnI and cTnT, a relevant example is a genome wide association study by Walsh et al. [47] of a large general population (19,500 Individuals) with the goal of providing insights into the mechanisms of elevations of troponin levels, the role of elevations in cardiovascular disease. Clinical outcomes were determined over a nearly 8-year follow-up period. The studies identified correlations with CVD and poor outcomes with single nucleotide polymorphisms (SNPs) in genes associated with the integrin signaling cascade involved in membrane stability as detailed in our earlier review and discussed further below. Importantly the SNPs were correlated differently with elevated serum cTnT and cTnI. This cutting-edge knowledge of regulation of membrane stability provides an example of how mechanistic understanding may lead to development therapeutic approaches. Therapeutic approaches with a goal to restore membrane integrity and cell viability by stabilization of sarcolemma integrity are well-described and under investigation. In reports discussing membrane stabilization employing synthetic block co-polymers emphasize the reliance of the design of this chemical based approach on the detailed molecular, mechanistic information [48,49].

Obesity, which is a well-documented co-morbidity in COVID-19 patients is another example where the connection between hyperlipidemia and elevations in serum cTnI and cTnT may not be considered an important biomarker. Arguments and data presented above dispute this. Little has been stated in the literature regarding the cytokine storm, myocarditis, and the mechanism for the rise in serum troponins. The evidence presented here and elsewhere of a relation between viral infections and programmed cell death not only informs the rationale of determination of serum troponins in COVID-19 patients, but also provides a path for development on new therapies. The need for new directions in therapeutic development is couched in the current use of dexamethasone for inflammation in the SARS-CoV-2 infections. In any case, serum troponins provide a measure of the effectiveness of anti-inflammatory agents with emphasis on myocarditis.

With advancements in vaccines and the current decline in cases of SARS-CoV-2 infections, there remains a need for understanding of mechanisms of elevations of serum troponins. Long term clinical issues are common in the COVID-19 patients and follow up with correlation of the troponin levels determined during hospitalization remains an important approach. One of many relevant examples is the cardiac dysfunction and clinical heart failure in COVID-19 patients with long term sequelae. There is poor understanding of whether long term disorders are related to myocarditis, hemodynamic instability, or microvascular disease. There is a need in this area of better diagnostic protocols, therapeutic strategies and development of new therapies [50]. In addition to the tools available, it is clearly important to have a better understanding of the mechanisms leading to damage of cardiac myocytes and resulting in elevated serum troponins.
